# EFFECTS OF A SIX-MONTH EXERCISE PROGRAM ON PHYSICAL ACTIVITY AMONG OLDER ADULTS WITH CHRONIC CONDITIONS

**DOI:** 10.1093/geroni/igac059.2891

**Published:** 2022-12-20

**Authors:** Revati Malani, Emily Erlenbach, Edward McAuley, Neha Gothe

**Affiliations:** University of Illinois--Urbana-Champaign, Urbana Champaign, Illinois, United States; University of Illinois, Urbana Champaign, Urbana Champaign, Illinois, United States; University of Illinois, Urbana Champaign, Urbana Champaign, Illinois, United States; University of Illinois at Urbana Champaign, Urbana, Illinois, United States

## Abstract

Health benefits of structured physical activity (PA) for older adults have been well documented. Older adults are highly impacted with major chronic conditions, leading to limitations in daily activities. This study examined the effects of an exercise program on moderate-vigorous physical activity (MVPA) and sedentary time after six months of exercise. Participants from a larger-ongoing trial were randomized into either yoga, aerobic or stretch-tone groups. A subsample of 58 participants was classified as chronic individuals (CI) based on presence of at least one chronic condition, and 31 as healthy individuals (HI), who completed accelerometer measures before and after the exercise program. A repeated measures analysis was performed to identify changes in PA over time for HI and CI. At both time-points, MVPA was significantly higher for HI. There was a significant increase in MVPA at month six for all participants, irrespective of health status. Mean MVPA in minutes for baseline HI: 153, CI: 98 and for month 6 HI: 188 CI: 116. There were no significant differences in sedentary time after the six-months of exercise. Although there was an increase in MVPA for HI and CI after structured exercise, no change was noted for sedentary time. Increased sedentary time has been proven to be a risk factor for poor health and although meeting criteria for MVPA is important, it is essential to address sedentary time in CI by implementing educational and interventional components that focus specifically on sedentary time. Funding: This data is from NCT04323163 funded by NIH-NIA – grant-AG066630.

